# An Analytical Target Profile for the Development of an In Vitro Release Test Method and Apparatus Selection in the Case of Semisolid Topical Formulations

**DOI:** 10.3390/pharmaceutics16030313

**Published:** 2024-02-23

**Authors:** Réka Szoleczky, Anita Kovács, Szilvia Berkó, Mária Budai-Szűcs

**Affiliations:** 1 Egis Pharmaceuticals Plc., Laboratory of Finished Product Analytical Development 3, Bökényföldi Str. 116-120, 1165 Budapest, Hungary; szoleczky.reka@egis.hu; 2 Institute of Pharmaceutical Technology and Regulatory Affairs, University of Szeged, Eötvös Str. 6, 6720 Szeged, Hungary; gasparne.kovacs.anita@szte.hu (A.K.); berko.szilvia@szte.hu (S.B.)

**Keywords:** analytical life cycle management, Analytical Target Profile (ATP), in vitro release test (IVRT), initial technology selection, target measurement uncertainty (TMU), diclofenac sodium

## Abstract

This study focuses on how to define an Analytical Target Profile (ATP) which is intended for use in practice and on facilitating the selection of in vitro release test (IVRT) technology for diclofenac sodium topical hydrogel and cream. The implementation involves incorporating the new draft guidelines of the International Council for Harmonisation (ICH Q14) and USP (United States Pharmacopeia) Chapter 1220. Four IVRT apparatuses were compared (USP Apparatus II with immersion cell, USP Apparatus IV with semisolid adapter, static vertical diffusion cell, and a new, in-house-developed flow-through diffusion cell) with the help of the ATP. Performance characteristics such as accuracy, precision, cumulative amount released at the end of the IVRT experiment, and robustness were investigated. We found that the best apparatus for developing IVRT quality control (QC) tests in both cases was USP II with an immersion cell. All four different IVRT apparatuses were compared with each other and with the data found in the literature.

## 1. Introduction

In vitro drug release tests (IVRTs) are one of the most important tools for drug development and the evaluation of semisolid dosage forms’ performance. The development of the drug release test as an analytical method poses a significant challenge for analysts. The new version of the ICH (International Council for Harmonisation) guidelines were adopted on 14 December 2023; ICH Q14 on analytical procedure development (Step 5) and the ICH Q2(R2) on the validation of analytical procedures (Step 5) are particularly relevant to our study. Together, the new ICH Q14 and the revised ICH Q2(R2) documents outline the proposed analytical development procedure and its validation for the analytical life cycle management approach. “The goal of development is to obtain an analytical procedure fit for its intended purpose: to measure an attribute or attributes of the analyzed material with the needed specificity/selectivity, accuracy and/or precision over the reportable range” [1]. In the new ICH Q14 analytical procedure development guidelines, two types of approaches are discussed: minimal (traditional) and enhanced. The result of the traditional trial-and-error approach is that the quality of the product is ensured by final product testing, which is confirmed by one-time validation. In contrast, the enhanced approach integrates elements of Quality by Design (QbD) and describes a science- and risk assessment-based analytical development procedure to ensure that the product fit for its intended use. The three stages of the analytical procedure life cycle are procedure design, procedure performance qualification, and continued procedure performance verification. The first stage of the analytical procedure life cycle includes knowledge gathering, establishing the ATP, understanding the effects of various process parameters on procedure performance, optimizing them, and defining the initial control strategy. An important element of the improved approach is the ATP, which outlines the intended purpose of the method development. It summarizes the expected performance characteristics, including the allowable error for the measurement, along with associated performance criteria for the analytical procedure. The ATP is independent of any specific analytical procedure; therefore, after defining the ATP, the analyst should choose the analytical technique(s) [1,2,3,4].

The Static Vertical Franz-type diffusion cell, USP Apparatus II with immersion cell, and USP Apparatus IV with semisolid adapter (SSA) are well-known apparatuses for in vitro performance testing of semisolid drug products, as described in USP Chapter 1724 [5]. However, the flow-through diffusion cell (FTDC) is not included in this chapter. Bronaugh and Stewart developed and described the flow-through diffusion cell apparatus in 1985 for percutaneous absorption studies [6]. In terms of application, the flow-through diffusion cell is not as widespread as the static Franz cell. This can be explained by various studies that aimed to compare static and flow-through diffusion cells, revealing similar absorption profiles and quantitative values for the different activities in both types of cells [6,7,8]. One advantage of using a flow-through diffusion cell is its ability to model the dynamics of blood flow, and maintaining sink conditions is more easily achieved [7,8]. Although, a drawback of the measurement is the challenge of ensuring the leak-free assembly of the device and preventing air being trapped between the membrane and the medium [7].

**Figure 1:**
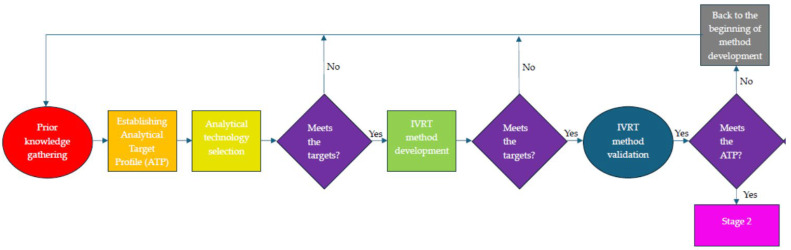
Flowchart of stage 1 of the analytical procedure life cycle.

This study focuses on defining the ATP for practical use and facilitating the selection of IVRT technology for topical hydrogels and creams containing diclofenac sodium (which are used as model products). This involves implementing the ICH Q14 draft guidelines [1] and USP (United States Pharmacopeia) Chapter 1220 [3]. In this article, we aim to compare four IVRT apparatuses (USP Apparatus II with immersion cell, USP Apparatus IV with semisolid adapter, static vertical diffusion cell, and a new, in-house-developed flow-through diffusion cell) with the assistance of the ATP, and to select a proper IVRT apparatus and method for further measurements using a minimum set of investigations. After selecting the considered apparatus for IVRT, the analytical method development phase can begin, followed by validation of the method (Figure 1). However, this is not within the scope of this article.

## 2. Materials and Methods

### 2.1. Chemicals and Materials

Diclofenac sodium salt, sodium chloride, di-sodium hydrogen phosphate dihydrate, and sodium hydroxide were obtained from Molar Chemicals Ltd. (Halásztelek, Hungary). Hypromellose (HPMC), polysorbate 60, castor oil, white petrolatum, cetostearyl alcohol, methylparaben, and propylene glycol were provided by Hungaropharma Ltd. (Budapest, Hungary).

Potassium dihydrogen phosphate was purchased from Thomasker (Budapest, Hungary). The ELGA PURELAB Chorus 1 lab water purification system was used to purify the water for the experiments (ELGA LabWater Headquarters, Lane End, UK).

Orthophosphoric acid was acquired from Merck (Darmstadt, Germany). Methanol (HPLC gradient grade) was purchased from Honeywell International Inc. (Charlotte, NC, USA).

All chemicals were of analytical grade. The receptor medium consisted of pH 7.4 phosphate-buffered saline (PBS) and pH 7.9 PBS containing 8 g sodium chloride, 0.19 g potassium dihydrogen phosphate, and 1.18 g di-sodium hydrogen phosphate dihydrate dissolved in 1000 mL distilled water. The solution was adjusted to pH 7.4 ± 0.05 with 85% orthophosphoric acid or pH 7.9 ± 0.05 with 1 M sodium hydroxide. pH 7.4 PBS and pH 7.9 PBS buffers were freshly prepared before the IVRT.

For the IVRT, we employed artificial MCE (a mixture of nitrocellulose and cellulose acetate) membrane filters with a diameter of 25 mm and a pore size of 0.22 µm, as well as artificial MCE membrane filters with a diameter of 47 mm and a pore size of 0.22 µm. They were provided by Labex Ltd. (Budapest, Hungary). Before each IVRT measurement, the MCE membrane filters were soaked in the receptor medium for 30 min, and an infinite dosage was applied during every IVRT measurement (approximately 300–700 mg). All IVRT samples were measured using Ultra-High Performance Liquid Chromatography (UHPLC), and the analytical method is described in Section 2.2.5.

### 2.2. Methods

The UHPLC measurements and evaluation of sample concentrations were performed using Empower 3 software (copyright 2010 Waters Corporation, Milford, MA, USA). Drug release rates for each IVRT method were calculated using Microsoft^®^ Excel^®^ Office 365, following the guidelines outlined in USP Chapter 1724 [5].

#### 2.2.1. Static Vertical Diffusion Cell (Franz Cell)

The vertical diffusion cell system (Teledyne Hanson Co., Chatsworth, CA, USA), containing 6 cells (diffusional surface area: 1.767 cm^2^) and equipped with an autosampler (Hanson Microette Autosampler System), was used to model the in vitro drug release from diclofenac sodium topical hydrogel and cream. Approximately 320 mg of the drug product was placed onto the 25 mm diameter cellulose membrane with a pore size of 0.22 µm (Labex Ltd., Budapest, Hungary). A receptor medium of 7 mL, with a pH of 7.4 ± 0.05 or pH 7.9 ± 0.05 PBS, was chosen and maintained at 32 ± 0.5 °C during the measurements. The stirring rate was set to 400 rpm. Samples of the acceptor medium (800 μL) were collected at 15, 30, 45, 60, 90, 120, 150, 180, 240, 300, and 360 min and then analyzed using UHPLC. The volume of the replacement medium was 1.1 mL of pH 7.4 PBS or pH 7.9 PBS.

#### 2.2.2. USP Apparatus II with Immersion Cell (USP II)

The next apparatus employed in our study was the USP Apparatus II dissolution test system (Vision^®^ G2 Elite 8, Teledyne Hanson Co., Chatsworth, CA, USA) with a 0.53 mL immersion cell (Teledyne Hanson Co., Chatsworth, CA, USA). The use of this cell (Model B) is described in USP Chapter 1724 [5]. The immersion cell, with a membrane surface of 1.77 cm^2^, containing diclofenac cream or hydrogel (size of sample: 600–700 mg), was placed in a 150 mL flat-bottom vessel. The 150 mL receptor medium at pH 7.4 PBS or pH 7.9 PBS was applied at 32.0 ± 0.5 °C. The assembly of the immersion cell is described in the Hanson Research Corporation user guide (with Small Volume Vessel) [9]. The mini spin-paddle stirrers were set to stir at 250 rpm. Samples of 1.0 mL were taken from the acceptor medium at 15, 30, 45, 60, 90, 120, 150, 180, 240, 300, and 360 min time points. No medium replacement occurred, as this had been accounted for during the calculation of the IVRT results.

#### 2.2.3. USP Apparatus IV (USP IV): Flow-Through Cell with 0.4 mL Semisolid Adapter

As the third system, USP Apparatus IV was utilized to model the in vitro drug release from topical hydrogel and cream containing diclofenac sodium. The USP Apparatus IV dissolution system (Sotax CE7 smart with CY 7 piston pump, Sotax Corporation, Westborough, MA, USA) with a 0.4 mL semisolid adapter was described in an earlier study [10]. Tablet cells of 22.6 mm with 1 mm glass beads and 400 µL SSA (diffusional surface area: 1.54 cm^2^) were used. We chose 11 sampling points (15, 30, 45, 60, 90, 120, 150, 180, 240, 300, and 360 min) to monitor the in vitro release rate (IVRR) of diclofenac sodium. This apparatus operated an open-loop configuration with flow rates of 2 and 4 mL/min (pulse rate: 20 pulses per minute) at 32 °C.

#### 2.2.4. Flow-Through Diffusion Cell (FTDC)

Our flow-through diffusion cell, uniquely designed in our laboratory and equipped with a syringe pump, is an open-system diffusion cell suitable for measuring in vitro diffusion and skin penetration. This apparatus features only one measuring block, a spiral diffusion cell with a volume of 875 μL, allowing for one parallel measurement at a time. To ensure the leak-free assembly of the device, a 0.22 µm cellulose membrane with a 15.5 mm diameter and a 1.76 cm^2^ diffusional surface area^,^ (Labex Ltd., Budapest, Hungary) was applied for the measurements. The IVRT time spanned 6 h, with eleven fractions of the pH 7.4 PBS receptor fluid being manually collected at 15, 30, 45, 60, 90, 120, 150, 180, 240, 300, and 360 min, respectively, and then measured by UHPLC. The flow rate under the membrane was adjusted to approximately 2 mL/min and 4 mL/min, with the system temperature set to 32 °C.

#### 2.2.5. Description of the UHPLC Method

The Acquity UPLC I-Class (Waters Corporation, Milford, MA, USA) was employed to analyze the IVRT samples, and the method was previously detailed in our earlier study [10]. The eluent used was a mixture of 20 mM pH 2.5 potassium dihydrogen phosphate buffer and methanol (MeOH) was used in a ratio of 36/64 (*v*/*v*), utilizing an isocratic elution mode. The flow rate was set to 0.45 mL/min, and the column used was the Acquity UPLC BEH UHPLC column with dimensions 2.1 mm × 50 mm, 1.7 µm, 130 Å (Waters Corporation, Milford, MA, USA), maintained at a temperature of 40 ± 0.5 °C. The UHPLC method had a run time of 3 min. The measurement and evaluation were conducted at a wavelength of 275 nm. For calibration, the injection volume was 2 µL, but during the measurement of the IVRT samples, different volumes were used depending on the diclofenac sodium concentration in the samples (2.0 µL in the case of flow-through diffusion cell, USP apparatus IV with semisolid adapter, and USP apparatus II with immersion cell; 0.5 µL from cream IVRT sample and 0.2 µL from hydrogel IVRT sample for the Franz-type diffusion cell).

#### 2.2.6. Establishing Analytical Target Profile (ATP)

The initial phase of the analytical method development involved the establishment of the ATP for the IVRT measurement. It needs to be established prior to starting the activities related to method development. As defined, “An ATP consists of a description of the intended purpose, appropriate details on the product attributes to be measured and relevant performance characteristics with associated performance criteria” [1]. The ATP should encompass the definition of the analyte and the product, including details such as dosage form, strength, matrix components, and route of administration. It should also cover aspects like range, acceptable bias, and precision (maximum allowable combined bias or target measurement uncertainty) [1,3]. “Target measurement uncertainty (TMU) is the maximum acceptable uncertainty in the reportable result that must be achieved by the analytical procedure” [11]. “Once the acceptable probability of making an incorrect decision of compliance has been established and a decision rule has been defined, the target measurement uncertainty is decided” [12].

The In Vitro Release Test Studies for Topical Drug Products Submitted in ANDAs draft guidance [13] and draft guidelines on quality and equivalence of topical products [14] helped us to set up the ATP. In this study, diclofenac sodium hydrogel and cream were used as model products.

## 3. Results

### 3.1. Definition of ATP for the IVRT

The first step of the analytical procedure life cycle approach is to define the ATP (Table 1) for the IVRT method for the development of the topically used diclofenac sodium hydrogel and cream, as suggested by the ICH Q14 draft guidelines and USP 1220 [1,3]. Both magistral semisolid products were prepared in our lab. The intended purpose of the development of the analytical method was the quantification of the diclofenac sodium API (active pharmaceutical ingredient) for an IVRT quality control (QC) test. The dosage strengths were 1% and 2%. Both products were stored in plastic containers with caps at 4–5 °C until the investigation. The matrix of the topical hydrogel containing diclofenac sodium included purified water, HPMC (hydroxypropyl methylcellulose), and propylene glycol. The excipients of the cream that contained diclofenac were cetostearyl alcohol, castor oil, polysorbate 60, methylparaben, and white petrolatum. Assuming a normal distribution, the calculation of TMU (σ) can be described using the following formula [12]:

(1) $$\sigma =\frac{x-µ}{z}=\frac{77\%-70\%}{1.65}=4.24\%,\;\;\;ifQ_{final}=70\%$$

where x is the upper specification limit of accuracy (in our case, it was 110%, so with 70% of Q_final_, x value is 77%), µ is the true value (100%), and z is the coverage factor (two-tailed, 90% confidence level 1.65). After the IVRT apparatus was chosen and the initial analytical control strategy (ACS) was established at the end of stage 1, it should be confirmed that the reportable values of the developed analytical procedure meet the ATP criteria [3]. If the measured results do not follow a normal distribution, a re-examination is required at the end of stage 1, by which time there will be a sufficient amount of measured IVRT data for this purpose. Determining the distribution was not part of our study; our purpose was to identify the IVRT apparatus and method with the minimal number of experiments, guided by the ATP.

The linearity and the robustness criteria in “The In Vitro Release Test Studies for Topical Drug Products Submitted in ANDAs” draft guidance [13] were used to establish the target of the ATP. The release of the API from a semisolid product can be considered linear “if the release rate should have an R^2^ value ≥ 0.97 across the recommended IVRT study duration of 4–6 h” [13]. This guidance describes the robustness test: “the IVRT method may be considered robust to a variation in the test method if the average slope of an IVRT run under the altered IVRT method parameters is within ± 15% of the average slope of the precision and reproducibility IVRT runs” [13].

The draft guidelines on the quality and equivalence of topical products described that “the duration of IVRT should be sufficient to characterize the release profile, ideally at least 70% of the active substance applied is released”, therefore we chose Q_final_ ≥ 70% as the target for the ATP [14]. According to the ICH Q2(R1) recommendations, the range of the quality requirements is a band widened by ± 20% [15].

After establishing the crucial ATP, which includes relevant performance characteristics with the associated performance criteria, the second step involved the initial technology selection for IVRT method development.

### 3.2. The Selection of the IVRT Technology

The ATP should lead the selection of analytical technology. In our case, multiple analytical technologies for IVRT were available in our laboratory, offering a choice from among four IVRT technologies (static vertical diffusion cell, USP Apparatus II with immersion cell, USP Apparatus IV with semisolid adapter, and a flow-through diffusion cell). This study focused on selecting the IVRT method based on the ATP. The UHPLC measurement technique and method were chosen; however, the UV spectrophotometric analytical method could not be used due to the UV active-matrix component. With the help of the UHPLC method, we measured the IVRT samples.

If the development of the product and the analytical method development proceed simultaneously, a wealth of measurement data becomes available for selecting the analytical technology. In our case, there is minimal prior knowledge of the IVRR of diclofenac sodium from the hydrogel and cream matrix. Before the preliminary IVRT experiment, the membrane inertness and the desired medium sink conditions of the receptor medium should be confirmed [13,14]. The membrane inertness test with MCE cellulose membrane was previously completed in our earlier study, where it was proved that the ME cellulose (mixed cellulose ester, nitrate, and acetate) membrane did not bind to diclofenac sodium, yielding a result of 100.1 ± 3.7% [10]. The criteria of sink conditions require a minimum three-times-higher concentration than the maximum concentration of the API in the receptor medium [14]. In our situation, the IVRT method parameters of the USP II with immersion cell, USP IV with SSA, and the static vertical diffusion cell (Franz cell) meet the sink condition criterion, but the FTDC does not (Table 2). Although, we must note that the inertness of the membrane itself and the sink condition may have different significance for different devices. However, in order to make the comparison, the inertness of the membrane and sink condition must be established as basic conditions. In the case of the sink condition, although it should be noted that the volume of the receptor phase is, in most cases, a basic apparatus property, in our case only the FTDC could not fulfill this condition at the starting point of the IVRT. Nevertheless, in the case of FTDC (and USP IV with SSA), sustaining the continuous replacement of the fresh receptor medium ensures the correct sink conditions more easily throughout the experiment. Both flow-through cell apparatuses were used in an open-loop configuration. Confirmation of the sink conditions for different apparatus is presented in Table 2.

The “usual” IVRT parameters employed for the initial measurements involved using pH 7.4 PBS (without cosolvent or surfactant) for all four apparatuses.

#### 3.2.1. In Vitro Test Results of Cream Containing Diclofenac Sodium

Creams containing 1% and 2% of diclofenac sodium underwent investigation according to the IVRT method described in Section 2.2. Figures S1, S3, S5 and S7 display the cumulative drug release per unit area on a linear time scale, as well as the cumulative drug release per unit area plotted against the square root of time. The means, standard deviations, and relative standard deviations of IVRR and the fluxes of diclofenac sodium from creams are presented in Table 3 and Table 4. Detailed data can be found in the Supplementary Materials (Tables S1 and S5).

Ideally, 70% of the diclofenac sodium should be released from the cream during the IVRT measurement [14], but in our case, it did not occur within the typical 6 h timeframe (Table 3). Therefore, the measurement time was extended from 6 h to 12 h.

Robustness tests were performed at flow rates of 2 mL/min and 4 mL/min (FTDC and USP IV with SSA) and at pH 7.4 and 7.9 (Franz cell, USP II with immersion cell and USP IV with SSA) The nominal IVRT parameters were set at pH 7.4 and 4 mL/min (where interpretation is possible).

The difference between the average slope measured with the altered IVRT parameters and the one measured with the nominal IVRT method parameters cannot be greater than 15% [13]. These results showed (Table 5) that the Franz cell and USP II with immersion cell IVRT methods were robust. Although, FTDC and USP IV with SSA deviated by more than 15% from the mean IVRR and, therefore, were not considered robust.

**Figure 2:**
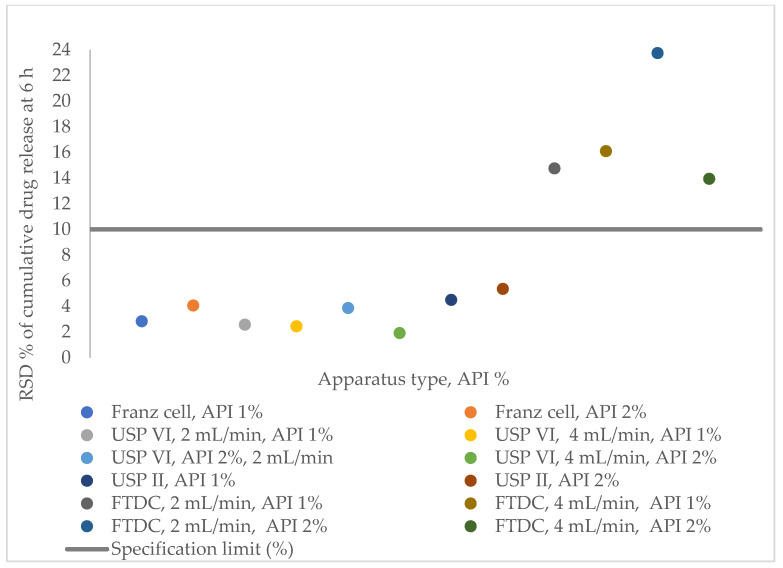
Relative standard deviation % of cumulative drug release of diclofenac sodium 1% and 2% creams at 6 h, measured with Franz cell, USP Apparatus II (with immersion cell), USP Apparatus IV (with SSA), and flow-through diffusion cell, in pH 7.4 receptor medium.

In the ATP, it was established that the method’s precision at the last sampling time point should be RSD (%) ≤ 10%. Figure 2 and Table 5 illustrate that the precision of the FTDC method does not meet the requirements outlined in the ATP, while the other apparatuses comply with the specification limit. For this reason, we did not carry out accuracy measurements with the FTDC apparatus. The lowest RSD (%) was observed with the USP Apparatus IV with SSA (1.91%).

The subsequent evaluation was an accuracy test conducted at a 100% nominal concentration using a 1% diclofenac sodium cream as reference. In our study, the IVRR of this cream at 6 h was ~30%. The accuracy measurements were carried out with diclofenac sodium pre-dissolved in pH 7.4 PBS medium and a placebo cream (cream without diclofenac sodium) was placed on the upper side of the MCE membrane filter. The accuracy test lasted for 6 h, with a single sample collected at the end of the test. The accuracy results, presented in raw data in Table S3 and summarized in Table 5, indicate that the investigations with the Franz cell, USP IV (with SSA), and USP II (with immersion cell) meet the performance criteria, which was described in the ATP (90–110%). Despite the adequacy of the Franz cell results, they were slightly lower than the accuracy observed with USP IV (with SSA) and USP II (with immersion cell). Because of this systematic error in the Franz cell measurements (bias ~−3.42), it is advisable to choose this apparatus for measuring diclofenac sodium cream in our specific case.

Based on preliminary measurements, the USP II with immersion cell apparatus seems to be the best choice to develop an IVRT analytical method for diclofenac sodium cream.

#### 3.2.2. In Vitro Test Results for the Hydrogel Containing Diclofenac Sodium

Hydrogels, containing 1% and 2% of diclofenac sodium, were also measured according to the IVRT method described in Section 2.2. The IVRT results showed that the Franz cell, USP II with immersion cell, and USP IV with SSA apparatuses met the targets established in the ATP. The drug release curves can be found in the Supplementary Materials (Figures S2, S4, S6 and S8). The means, standard deviations, and relative standard deviations of IVRR and the fluxes of diclofenac sodium from hydrogels are presented in Table 6 and Table 7. Detailed data can be found in the Supplementary Materials (Tables S2 and S6).

**Figure 3:**
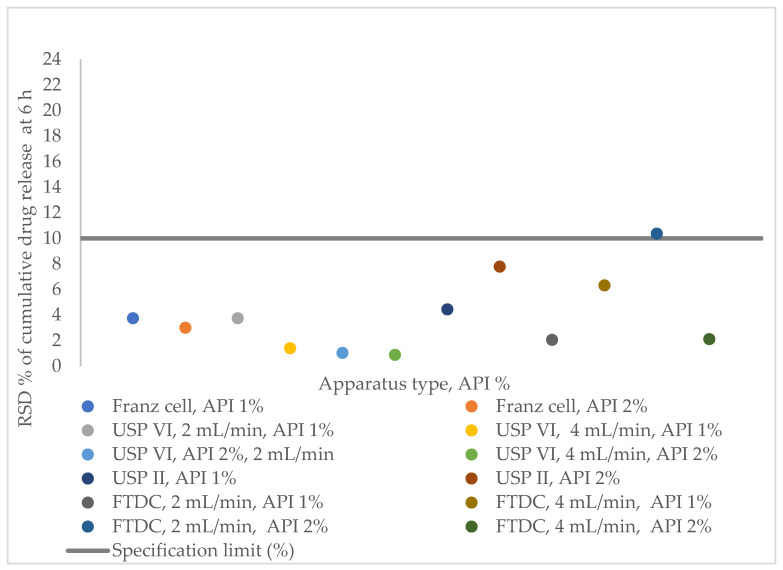
Relative standard deviation % of cumulative drug release of diclofenac sodium 1% and 2% hydrogels at 6 h measured with Franz cell, USP Apparatus II (with immersion cell), USP Apparatus IV (with SSA), and flow-through diffusion cell.

The results of the IVRT method with FTDC showed that the method was not robust with respect to pH changes, and under the FTDC setting of 2 mL/min, the API 2% method’s precision RSD% at the last timepoint exceeded 10% (Figure 3, Table 8). Although the accuracy test (at 100% nominal concentration with 1% diclofenac sodium hydrogel placebo; in our case the IVRR at 6 h was approximately 90%) passed the criteria described in the ATP, the result for the Franz cell was lower (96.58%) than for the other two apparatuses (USP II with immersion cell and USP IV with SSA). Therefore, the Franz cell is not likely to be the first choice for the development of the IVRT method in the case of a hydrogel that contains diclofenac sodium.

The USP II with immersion cell apparatus and/or USP IV with SSA seems to be the optimal choice for developing the IVRT analytical method for a hydrogel that contains diclofenac sodium based on preliminary measurements.

## 4. Discussion

The planning of in vitro drug release studies and the selection of the appropriate apparatus and method are crucial for characterizing pharmaceutical formulations. The study of drug release from a topical semisolid formulation serves as a clear indicator of the formulation’s performance. The developed IVRT method should be sufficiently robust, providing a well-reproducible measurement. Additionally, the developed IVRT method should be capable of detecting microstructural variations in the formulation.

Currently, several accepted apparatuses are available, including the Static Vertical Franz-type diffusion cell, USP Apparatus II with immersion cell, and USP Apparatus IV with semisolid adapter (SSA), which are official pharmacopeial apparatuses for performing IVRT measurements on semisolid formulations [5]. In contrast, the flow-through diffusion cell (FTDC) is a new apparatus developed in our laboratory. Its arrangement is similar to the Static Vertical Diffusion cell (Hanson), meaning that it is positioned towards the upper part of the cell and separated from the receptor phase by a membrane. However, unlike the Franz cell, the donor cell is open at the top. Another notable difference lies in the arrangement of the receptor phase: beneath the membrane, the medium circulates in a spiral path within the receptor cell, maintaining contact with the membrane throughout the spiral movement. This medium is continuously flowing, differing from the static medium in the Franz cell between samplings. FTDC holds an advantage over the static cell in that it allows continuous sampling and, with the continuous exchange of the receptor phase, effectively models blood flow in the skin. Additionally, maintaining the “sink condition” is easier with FTDC.

In our present work, we compared two static and two flow-through apparatuses, incorporating both open- and closed-cell configurations. This approach covered the most prevalent pharmacopeial apparatuses, along with a potential new arrangement type. The microstructure of semisolid formulations can pose a considerable challenge for reproduction. Furthermore, the employed apparatuses exhibit several unfavorable characteristics that complicate the reproducibility or evaluation of the IVRT method. All four apparatuses presented utilize membranes, and both the type and fitting of the membranes in the applied cells pose a potential source of error. Our exploration of membrane types in our testing systems is detailed in a prior article [10], and as such, we do not delve into it in the current publication.

Another significant drawback of the apparatuses used in studying the release of semisolid active ingredients pertains to the filling of the donor cell, which must accommodate the semisolid formulation. This process involves ensuring a leak-free assembly, achieving a perfect fit between the formulation, membrane, and receptor phase, and ensuring the absence of air bubbles. Moreover, it is paramount to take a note of distortions that may arise from the dead space within automatic samplers. Taking all these factors into account, developing a well-reproducible IVRT method for semisolid formulations proves challenging, as has been described in several instances in the international literature.

In the study by Liebenberg et al., it was found that the release rates of hydrocortisone, salicylic acid, ascorbic acid, and triclosan from creams and gels were slower when measured with the flow-through cells compared to measurements with the Franz cell and immersion cells [17]. Tanja Ilić et al. came to the same results, finding total agreement between the results obtained with the Franz cell and immersion cells [18]. Similarly, in our study, the investigations suggested that the IVRRs of a cream which contains diclofenac sodium (µg × cm^−2^ × min^−0.5^) were the lowest when measured with FTDC and USP IV (with SSA). Those measured by USP II (with immersion cell) and Franz cell were consistent, and they were 1.3 times higher than those measured with FTDC. The ranking of the IVRR (µg × cm^−2^ × min^−0.5^) for our creams was: USP IV, SSA ≈ FTDC < USP II, immersion cell ≈ Franz cell.

Sanghvi and Collins compared the cumulative release rates of an enhancer (immersion cell) and Franz cell measured with hydrocortisone 1% ointment. It was found that the cumulative release using a cellulose membrane was more than threefold higher with the enhancer cell (immersion cell) compared to that seen with the Franz cell [19]. In contrast, in our study, concerning a cream which contains diclofenac sodium, the IVRR measured with USP II (immersion cell) and Franz cell was the same.

Bao et al. compared the reproducibility and discrimination ability of three apparatus types (USP Apparatus IV with semisolid adapters, USP Apparatus 2 with enhancer cells, and Franz diffusion cells) using ophthalmic ointment preparations The coefficient of variation (CV%) of the drug release rate indicated no significant differences among the three methods, and our results also confirmed this [20].

In terms of the diclofenac sodium hydrogel, the IVRR (µg × cm^−2^ × min^−0.5^) measured by USP IV (with SSA) was the highest, while those measured by USP II (with immersion cell) and the Franz cell were the same. The rank order of diclofenac sodium’s IVRR (µg × cm^−2^ × min^−0.5^) from the hydrogel is: USP II, immersion cell ≈ Franz cell < FTDC <USP IV, SSA. Clowes et al., investigated the absorption of water and mannitol through human, pig, and rat epidermises measured with static diffusion cell and flow-through diffusion cell. Contrary to our results, their findings showed that flow-through diffusion cells and static diffusion cells were similar in their ability to measure the percutaneous absorption of water-soluble penetrants [7]. M.J. Lucero et al. used Franz cells and enhancer cells to investigate the controlled drug release of theophylline from a three-dimensional gel structure. They concluded that the Enhancer Cell apparatus provided more reliable and reproducible results than the Franz diffusion cell due to better monitoring and control of different variables. We also concur with this observation [21]. The rank of the IVRRs (µg × cm^−2^ × min^−0.5^) of our hydrogels was: USP II, immersion cell ≈ Franz cell < FTDC < USP IV, SSA. The present work aimed to establish an ATP for the IVRT quality control test for a diclofenac sodium cream and hydrogel based on the new ICHQ14 draft guidelines [1] and USP Chapter 1220 [3]. Following the definition of the ATP, which was informed by the In Vitro Release Test Studies for Topical Drug Products Submitted in ANDAs draft guidance [13], draft guidelines on quality and equivalence of topical products [14], and ICH Q2(R1) Validation of Analytical Procedures: Text and Methodology guidelines [15], our next step was the initial selection of technology for the development of the IVRT method. We could choose from four different IVRT apparatuses (FTDC, USP II with immersion cell, USP IV with 0.4 mL SSA, and static vertical diffusion cell). The HPLC method used for measuring IVRT samples was determined based on its compatibility with the matrix components, avoiding interference.

Two very common semisolid topical forms were examined in our study: o/w cream and hydrogel. In both formulations, the active ingredient was present in a dissolved state. The components of the formulated ATP assist in the selection of an appropriate IVRT method for the specific formulation. Our findings clearly indicate that each drug form exhibits distinct precision characteristics among the four apparatuses, and even within the same device, the precision may vary between the two drug forms. In terms of the precision of both of the semisolid systems, the USP IV, SSA apparatus proved to be the most favorable, while the FTDC was the least favorable. For the latter apparatus, the precision of the cream system exceeded the limit specified in the ATP (Table 5), making it unsuitable as a reproducible measurement method for creams. However, for the hydrogel form, the precision was significantly improved with this apparatus. This substantial difference is likely explained by the formation of a poorly reproducible formulation–membrane–receptor interface, which is dependent on the formulation type, especially for a donor phase which is in the open position at the top.

The appropriate selection of the duration of the IVRT method is important for several reasons. It is necessary to specify a test duration that is sufficiently long to adequately characterize the kinetics of drug diffusion, while also preventing degradation processes that could negatively impact the drug or the carrier system, leading to the potential misinterpretation of results. In accordance with the EMA draft guidelines, it is recommended that, at the last measurement point, a minimum of 70% of the drug should have diffused, and the ascending linear phase should include at least six measurement points. The 70% drug release was achieved in the hydrogel forms for all apparatuses, but in the cream forms, it notably fell short of this value. Interestingly, both the Franz cell and FTDC exhibited considerably higher values at the last measurement point compared to the other two cells, even though they had a smaller receptor volume. It is worth noting that, in the case of the FTDC, the sink conditions could not be met at the starting point and were only met during dynamic flow. A possible explanation for this phenomenon with these two cell types is the dynamics of receptor phase exchange. With the FTDC, the receptor medium continuously flows beneath the membrane, facilitating the renewal of the membrane–receptor phase interface. In the context of the Franz cell, although the cell remains static during sampling, a volume greater than the actual sample is exchanged in the cell’s receptor medium. This exchange occurs as part of the cleaning of the automatic sampling system’s tubing, leading to the renewal of the receptor surface under the membrane. This interface renewal may be exploited for more structured, slower drug release systems, achieving satisfactory results within a relatively shorter test duration.

The accuracy of the method is also a critical parameter, and, for this purpose, we specified a specification limit between 90–110% in our ATP table. Since FTDC was no longer suitable in terms of precision, we only performed this test for the remaining three apparatuses using drug-free blank formulations. All three apparatuses met the criterion, showing no deviation between the cream and hydrogel systems. However, regarding the Franz cell, we observed a more significant, but still within the limit, decrease. This decrease may be attributed to the systematic error of the mentioned apparatus or the back diffusion of the receptor medium towards the donor chamber during sampling.

The robustness regarding pH was evaluated on three apparatuses (USP II, IV, and Franz cell), while the flow rate was characterized by the flow rate within two apparatuses (USP IV, SSA, and FTDC). In terms of pH robustness, significant differences were clearly observed, particularly for the cream systems, with the USP IV, SSA apparatus showing a deviation beyond the limit. The cause of this is not currently identifiable from the measurement data and device types; further measurements are needed to clarify this in the future.

The flow rate robustness for FTDC in cream systems cannot be justified; presumably, for this apparatus type, since the sink condition cannot be ensured with the chosen medium in the initial state, the drug release rate depends on the flow rate. Meanwhile, in the system where the sink condition is fulfilled (USP IV, SSA), the flow rate is less influenced, and the chosen range of 2 to 4 mL/min is robust for the measurements.

As a final conclusion, the results of the preliminary IVRT experiments of the cream which contains diclofenac sodium showed that the release of the diclofenac sodium from the cream matrix was slow, as measured by all four IVRT apparatuses, and the Q_final_ ≥ 70% criterion described in the ATP was not met. Therefore, the IVRT measurement should be at least 12 h long. Based on our results, the USP II with immersion cell apparatus will be the best choice to develop an analytical IVRT method for diclofenac sodium cream, and the USP II with immersion cell apparatus and/or USP IV with SSA is best for diclofenac sodium hydrogel.

## Figures and Tables

**Table 1:** ATP of IVRT of cream and hydrogel that contain diclofenac sodium.

Attributes	Target	Justification
Accuracy	90–110%	The procedure must be able to accurately quantify diclofenac sodium in IVRT samples in the range of 50% to 120% of the nominal concentration with accuracy and precision, ensuring that measurements fall within ± 4.24 of the true value with 90% probability.
Method Precision (measured at the last sampling point of the IVRT)	RSD (%) at the last time points ≤ 10%	
Linearity	R^2^ ≥ 0.97 [13]	
Range	±20% over the specified range [15]	
Cumulative amount released at the end of the IVRT experiment	Q_final_ ≥ 70% [14]	The duration of the IVRT should be sufficient to characterize the release profile, with data collected at six time points within the linear portion of the drug release profile. Ideally, at least 70% of the applied diclofenac sodium should be released [14].
Robustness	Mean slope (µg × cm^−2^ × min^−0.5^) of an IVRT run with pH 7.4 and pH 7.9 medium, or 2 mL/min and 4 mL/min, should fall within ± 15%	The average slope of the IVRR of diclofenac sodium, measured with a pH 7.4 and pH 7.9 medium and/or 2 mL/min and 4 mL/min (2 × 6 measurements), should not deviate by more than 15% from the nominal method parameter settings [13].

**Table 2:** Confirmation of the sink conditions for different apparatuses.

Parameters	USP II with Immersion Cell	USP IV with SSA	Franz Cell	FTDC
Maximum dosage strength (%)	2			
Volume of the medium (mL)	150	10.2	7	0.875
Maximum sample weight (mg)	720	500	400	300
Maximum concentration of the API in the receptor medium (mg/mL)	0.1	1.0	1.1	6.9
Sink condition (mg/mL)	0.3	2.9	3.4	20.6
Solubility of diclofenac sodium in pH 7.4 PBS medium (mg/mL)	8.2 ± 0.7 [16]			

**Table 3:** IVRRs and fluxes of diclofenac sodium from 1% and 2% cream measured for 6 h using different methods with pH 7.4 medium.

Apparatus		Franz Cell	Franz Cell	USP IV with SSA	USP IV with SSA	USP IV with SSA	USP IV with SSA	USP II with Immersion Cell	USP II with Immersion Cell	FTDC	FTDC	FTDC	FTDC
API (%)		1%	2%	1%	1%	2%	2%	1%	2%	1%	1%	2%	2%
pH		pH 7.4	pH 7.4	pH 7.4	pH 7.4	pH 7.4	pH 7.4	pH 7.4	pH 7.4	pH 7.4	pH 7.4	pH 7.4	pH 7.4
Flow rate (mL/min) or stirring speed (rpm)		400 rpm	400 rpm	2 mL/min	4 mL/min	2 mL/min	4 mL/min	250 rpm	250 rpm	2 mL/min	4 mL/min	2 mL/min	4 mL/min
IVRR at 6 h (%)	Mean	49.29	61.31	28.09	30.02	23.50	25.62	26.09	38.00	30.47	56.64	37.09	42.96
	SD	1.40	2.49	0.66	0.76	0.97	0.49	1.33	2.85	4.52	8.68	8.90	5.62
	RSD%	2.83	4.06	2.36	2.53	4.13	1.93	5.09	7.49	14.85	15.33	23.99	13.07
IVRR at 6 h (µg/cm^2^)	Mean	836.79	2081.77	878.38	954.04	1502.96	1628.51	940.41	2692.42	530.73	979.98	1274.83	1478.47
	SD	23.71	84.54	22.55	23.28	58.17	31.14	42.27	144.37	78.28	157.73	302.60	206.03
	RSD%	2.83	4.06	2.57	2.44	3.87	1.91	4.50	5.36	14.75	16.10	23.74	13.94
Flux (µg × cm^−2^ × min^−0.5^)	Mean	44.13	97.45	46.63	49.89	84.96	85.09	46.36	114.52	25.67	50.98	70.37	88.60
	SD	2.10	4.34	1.24	1.99	3.61	1.56	2.07	11.59	1.15	7.31	18.47	14.92
	RSD%	4.76	4.46	2.66	3.98	4.25	1.83	4.46	10.12	4.47	14.33	26.25	16.84

**Table 4:** IVRRs and fluxes of diclofenac sodium from 1% and 2% creams measured for 6 h using different methods with pH 7.9 medium.

Apparatus		Franz Cell	Franz Cell	USP IV with SSA	USP IV with SSA	USP II with Immersion Cell	USP II with Immersion Cell
API (%)		1%	2%	1%	2%	1%	2%
pH		pH 7.9	pH 7.9	pH 7.9	pH 7.9	pH 7.9	pH 7.9
Flow rate (mL/min) or stirring speed (rpm)		400 rpm	400 rpm	4 mL/min	4 mL/min	250 rpm	250 rpm
IVRR at 6 h (%)	Mean	43.49	56.79	26.32	28.01	25.06	35.55
	SD	3.91	1.66	1.05	0.98	1.14	1.39
	RSD%	9.00	2.93	3.99	3.52	4.54	3.91
IVRR at 6 h (µg/cm^2^)	Mean	728.88	1928.25	812.62	1782.17	892.44	2442.54
	SD	23.97	56.53	25.98	67.52	59.92	83.34
	RSD%	3.29	2.93	3.20	3.79	6.71	3.41
Flux (µg × cm^−2^ × min^−0.5^)	Mean	38.02	96.78	41.94	93.44	44.38	111.15
	SD	1.42	4.55	1.77	3.55	3.22	5.25
	RSD%	3.73	4.70	4.23	3.79	7.26	4.73

**Table 5:** Predefined acceptance criteria and results for 1% and 2% diclofenac cream in the selection of the IVRT method.

Apparatus	Attributes	Target	Results of 1% Diclofenac Cream		Results of 2% Diclofenac Cream	
Franz cell	Method precision	RSD (%) at the last timepoint ≤ 10%	2.83	passed	4.06	pass
USP II, immersion cell			4.50	passed	5.36	pass
USP IV, SSA			2.44	passed	1.91	pass
FTDC			16.10	failed	13.94	failed
Franz cell	The cumulative amount released at the end of the IVRT experiment	Q_final_ ≥ 70%	49.29	failed	61.30	failed
USP II, immersion cell			26.10	failed	38.00	failed
USP IV, SSA			30.02	failed	25.62	failed
FTDC			56.64	failed	42.96	failed
Franz cell	Accuracy	90–110%	96.58	passed	-	-
USP II, immersion cell			100.94	passed	-	-
USP IV, SSA			99.04	passed	-	-
Franz cell	Robustness (pH)	Mean slope of an IVRT run with pH 7.4 and pH 7.9 medium should be within ± 15%	−13.85	passed	−0.68	passed
USP II, immersion cell			−4.26	passed	−2.94	passed
USP IV, SSA			−15.93	failed	9.81	passed
USP IV, SSA	Robustness (flow rate)	Mean slope of an IVRT run with 2 mL/min and 4 mL/min flow rate should be within ± 15%	−6.55	passed	−0.15	passed
FTDC			−49.67	failed	−20.58	failed

**Table 6:** IVRRs and fluxes of diclofenac sodium from 1% and 2% hydrogel measured for 6 h using different methods with pH 7.4 medium.

Apparatus		Franz Cell	Franz Cell	USP IV with SSA	USP IV with SSA	USP IV with SSA	USP IV with SSA	USP II with Immersion Cell	USP II with Immersion Cell	FTDC	FTDC	FTDC	FTDC
API (%)		1%	2%	1%	1%	2%	2%	1%	2%	1%	1%	2%	2%
pH		pH 7.4	pH 7.4	pH 7.4	pH 7.4	pH 7.4	pH 7.4	pH 7.4	pH 7.4	pH 7.4	pH 7.4	pH 7.4	pH 7.4
Flow rate (mL/min) or stirring speed (rpm)		400 rpm	400 rpm	2 mL/min	4 mL/min	2 mL/min	4 mL/min	250 rpm	250 rpm	2 mL/min	4 mL/min	2 mL/min	4 mL/min
IVRR at 6 h (%)	Mean	92.39	91.74	98.95	94.23	98.65	100.57	86.47	86.86	92.87	104.61	85.70	98.74
	SD	4.24	3.85	4.08	1.20	0.93	0.80	2.89	7.71	1.91	6.21	7.55	1.44
	RSD%	4.59	4.20	4.12	1.28	0.94	0.80	3.35	8.87	2.06	5.94	8.81	1.45
IVRR at 6 h (µg/cm^2^)	Mean	1663.29	3592.50	3124.55	3034.97	6349.44	6477.20	3135.51	6208.33	1606.13	1828.62	2967.02	3417.46
	SD	62.26	107.65	116.85	42.52	66.03	57.16	139.02	483.03	32.95	115.51	307.51	72.24
	RSD%	3.74	3.00	3.74	1.40	1.04	0.88	4.43	7.78	2.05	6.32	10.36	2.11
Flux (µg × cm^−2^ × min^−0.5^)	Mean	162.46	341.57	268.11	297.09	570.50	626.25	177.72	346.22	172.95	184.59	333.76	438.73
	SD	9.12	8.55	3.89	22.54	31.26	17.37	12.19	34.92	2.67	13.90	51.15	56.42
	RSD%	5.61	2.50	1.45	7.59	5.48	2.77	6.86	10.08	1.54	7.52	15.33	12.60

**Table 7:** IVRRs and fluxes of diclofenac sodium from 1% and 2% hydrogels measured for 6 h using different methods with pH 7.9 medium.

Apparatus		Franz Cell	Franz Cell	USP IV with SSA	USP IV with SSA	USP II with Immersion Cell	USP II with Immersion Cell
API (%)		1%	2%	1%	2%	1%	2%
pH		pH 7.9	pH 7.9	pH 7.9	pH 7.9	pH 7.9	pH 7.9
Flow rate (mL/min) or stirring speed (rpm)		400 rpm	400 rpm	4 mL/min	4 mL/min	250 rpm	250 rpm
IVRR at 6 h (%)	Mean	96.94	99.06	98.14	99.46	87.97	88.65
	SD	3.48	1.80	3.30	1.12	1.63	1.96
	RSD%	3.59	1.81	3.36	1.13	1.85	2.21
IVRR at 6 h (µg/cm^2^)	Mean	1654.36	3382.65	3061.12	6392.06	3281.31	6665.85
	SD	51.02	117.73	42.60	93.57	163.59	500.31
	RSD%	3.08	3.48	1.39	1.46	4.99	7.51
Flux (µg × cm^−2^ × min^−0.5^)	Mean	155.46	329.37	286.21	566.84	185.93	376.85
	SD	15.24	13.82	10.53	39.10	11.19	27.30
	RSD%	9.80	4.20	3.68	6.90	6.02	7.24

**Table 8:** Predefined acceptance criteria and results for 1% and 2% diclofenac hydrogels in the selection of the IVRT method.

	Attributes	Target	1% Hydrogel Results		2% Hydrogel Results	
Franz cell	Method Precision	RSD (%) at the last timepoint ≤ 10%	3.74	passed	3.00	passed
USP II, immersion cell			4.43	passed	7.78	passed
USP IV, SSA			1.40	passed	0.88	passed
FTDC			6.32	passed	2.11	passed
Franz cell	The cumulative amount released at the end of the IVRT experiment	Q_final_ ≥ 70%	92.40	passed	91.74	passed
USP II, immersion cell			86.47	passed	86.86	passed
USP IV, SSA			94.23	passed	100.57	passed
FTDC			104.61	passed	98.74	passed
Franz cell	Accuracy	90–110%	94.64	passed	-	-
USP II, immersion cell			102.00	passed	-	-
USP IV, SSA			97.08	passed	-	-
Franz cell	Robustness (pH)	Mean slope of an IVRT run with pH 7.4 and pH 7.9 medium should be within ± 15%	−4.31	passed	−3.57	passed
USP II, immersion cell			4.62	passed	8.85	passed
USP IV, SSA			6.24	passed	−9.49	passed
USP IV, SSA	Robustness (flow rate)	Mean slope of an IVRT run with 2 mL/min and 4 mL/min flow rate should be within ± 15%	−0.48	passed	−8.90	passed
FTDC			−6.31	passed	−23.93	failed

## Data Availability

The data presented in this study are available on request from the corresponding author.

## Supplementary Materials

The following supporting information can be downloaded at: https://www.mdpi.com/article/10.3390/pharmaceutics16030313/s1, Figure S1: (a) Cumulative amount of diclofenac sodium penetrated through the MCE membrane plotted against time [min], (b) cumulative amount of diclofenac sodium penetrated through the MCE membrane plotted against square root of time [min^0.5^]. Instrument: Flow-through diffusion cell. Receptor medium: pH 7.4. Flow rate 2 and 4 mL/min. Product: Cream 1% and 2%. The data represent the mean ± standard error of the mean for five replicates. Figure S2: (a) Cumulative amount of diclofenac sodium penetrated through the MCE membrane plotted against time [min], (b) cumulative amount of diclofenac sodium penetrated through the MCE membrane plotted against square root of time [min^0.5^]. Instrument: Flow-through diffusion cell. Receptor medium: pH 7.4. Flow rate 2 and 4 mL/min. Product: Hydrogel 1% and 2%. The data represent the mean ± standard error of the mean for five replicates. Figure S3: (a) Cumulative amount of diclofenac sodium penetrated through the MCE membrane plotted against time [min], (b) cumulative amount of diclofenac sodium penetrated through the MCE membrane plotted against square root of time [min^0.5^]. Instrument: USP Apparatus IV with semisolid adapter. Receptor media: pH 7.4 and 7.9. Flow rate 2 and 4 mL/min. Product: Cream 1% and 2%. The data represent the mean ± standard error of the mean for six replicates. Figure S4: (a) Cumulative amount of diclofenac sodium penetrated through the MCE membrane plotted against time [min], (b) cumulative amount of diclofenac sodium penetrated through the MCE membrane plotted against square root of time [min^0.5^]. Instrument: USP Apparatus IV with semisolid adapter. Receptor media: pH 7.4 and 7.9. Flow rate 2 and 4 mL/min Product: Hydrogel 1% and 2%. The data represent the mean ± standard error of the mean for six replicates. Figure S5: (a) Cumulative amount of diclofenac sodium penetrated through the MCE membrane plotted against time [min], (b) cumulative amount of diclofenac sodium penetrated through the MCE membrane plotted against square root of time [min^0.5^]. Instrument: USP Apparatus II with immersion cell. Receptor media: pH 7.4 and 7.9. Product: Cream 1% and 2%. The data represent the mean ± standard error of the mean for six replicates. Figure S6: (a) Cumulative amount of diclofenac sodium penetrated through the MCE membrane plotted against time [min], (b) cumulative amount of diclofenac sodium penetrated through the MCE membrane plotted against square root of time [min^0.5^]. Instrument: USP Apparatus II with immersion cell. Receptor media: pH 7.4 and pH 7.9. Product: Cream 1% and 2%. The data represent the mean ± standard error of the mean for six replicates. Figure S7: (a) Cumulative amount of diclofenac sodium penetrated through the MCE membrane plotted against time [min], (b) cumulative amount of diclofenac sodium penetrated through the MCE membrane plotted against square root of time [min^−0.5^]. Instrument: Static Vertical diffusion cell (Franz cell). Receptor media: pH 7.4 and 7.9. Product: Cream 1% and 2%. The data represent the mean ± standard error of the mean for six replicates. Figure S8: (a) Cumulative amount of diclofenac sodium penetrated through the MCE membrane plotted against time [min], (b) cumulative amount of diclofenac sodium penetrated through the MCE membrane plotted against square root of time [min^0.5^]. Instrument: Static Vertical diffusion cell (Franz cell). Receptor media: pH 7.4 and 7.9. Product: Hydrogel 1% and 2%. The data represent the mean ± standard error of the mean for six replicates. Table S1: In vitro release rates and fluxes of diclofenac sodium from cream 1% and 2% at 6 h measured with different methods with pH 7.4 medium. Table S2: In vitro release rates and fluxes of diclofenac sodium from hydrogel 1% and 2% at 6 h measured with different methods with pH 7.4 medium. Table S3: Results of accuracy measurement at a nominal concentration of 100% using cream matrix. Table S4: Results of accuracy measurement at a nominal concentration of 100% using hydrogel matrix. Table S5: IVRRs and fluxes of diclofenac sodium from 1% and 2% cream measured for 6 h using different methods with pH 7.9 medium. Table S6: IVRR and fluxes of diclofenac sodium from 1% and 2% hydrogel measured for 6 h using different methods with pH 7.9 medium.
