# Supplementary material for: An Analytical Target Profile for the Development of an In Vitro Release Test Method and Apparatus Selection in the Case of Semisolid Topical Formulations
Source: Pharmaceutics. 2024 Feb 23;16(3):313. doi: 10.3390/pharmaceutics16030313 (PMC10975506; doi:10.3390/pharmaceutics16030313)
Supplement: Supplementary file 1 [file pharmaceutics-16-00313-s001.zip › pharmaceutics-2873772-supplementary.pdf]

pharmaceutics

Supplementary Materials

Analytical target profile for *in vitro* release test method development and apparatus selection in the case of semisolid topical formulations

Réka Szoleczky [1,2], Anita Kovács [2], Szilvia Berkó [2] and Mária Budai-Szűcs [2,*]

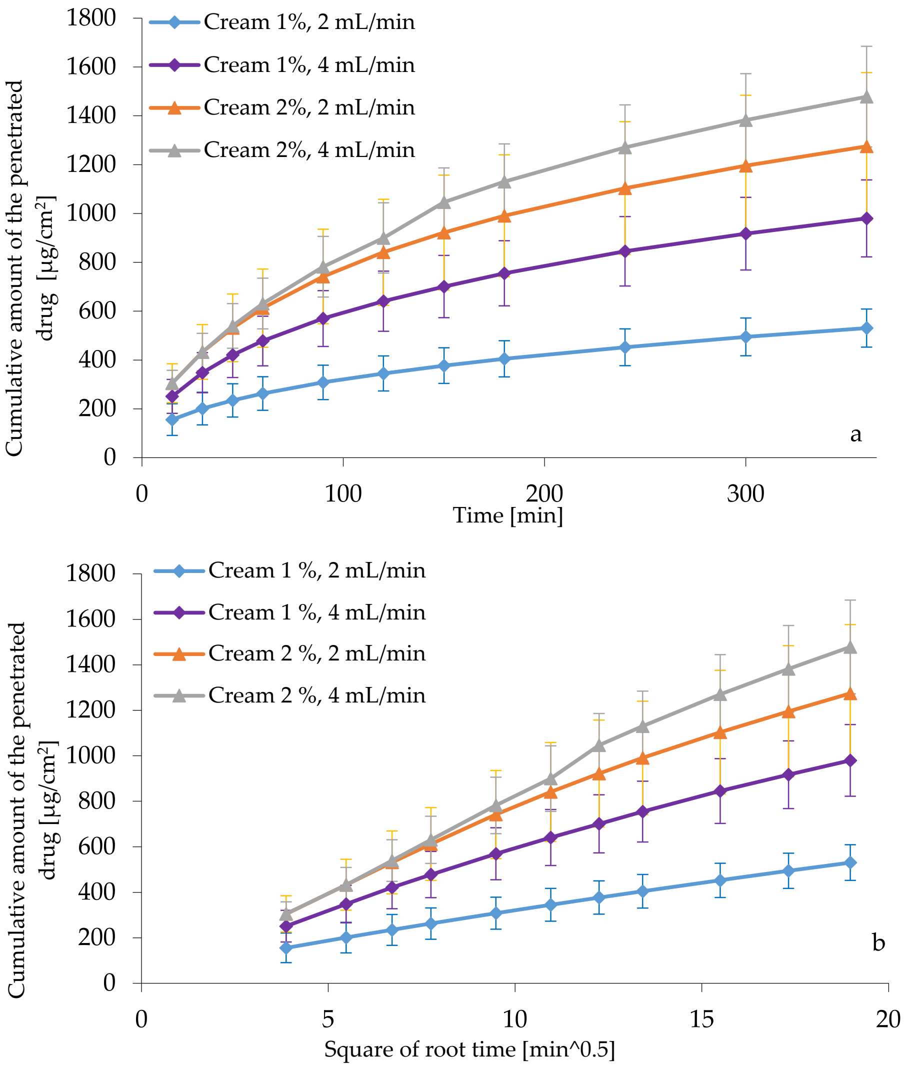

Figure S1. (**a**) Cumulative amount of diclofenac sodium penetrated through the MCE membrane plotted against time [min], (**b**) cumulative amount of diclofenac sodium penetrated through the MCE membrane plotted against square root of time [min^0.5]. Instrument: Flow-through diffusion cell. Receptor medium: pH 7.4. Flow rate 2 and 4 mL/min. Product: Cream 1 % and 2 %. The data represent the mean ± standard error of the mean for five replicates.

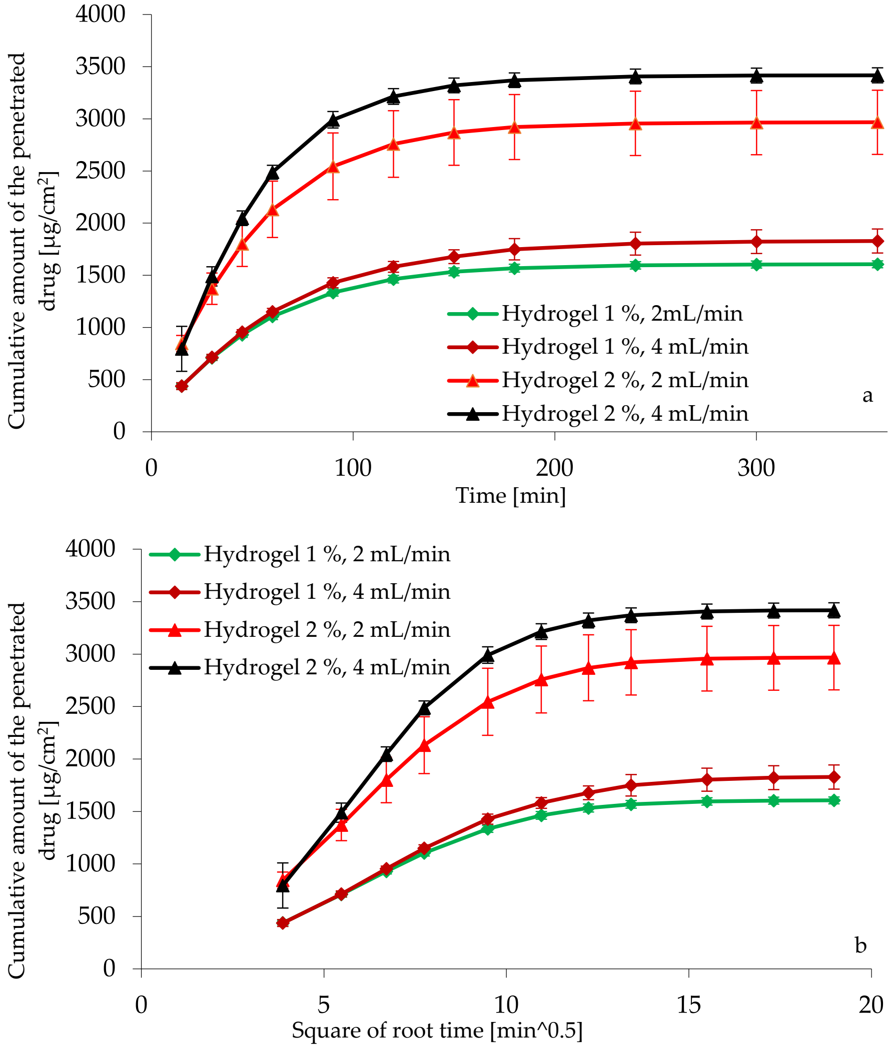

Figure S2. (**a**) Cumulative amount of diclofenac sodium penetrated through the MCE membrane plotted against time [min], (**b**) cumulative amount of diclofenac sodium penetrated through the MCE membrane plotted against square root of time [min^0.5]. Instrument: Flow-through diffusion cell. Receptor medium: pH 7.4. Flow rate 2 and 4 mL/min. Product: Hydrogel 1 % and 2 %. The data represent the mean ± standard error of the mean for five replicates.

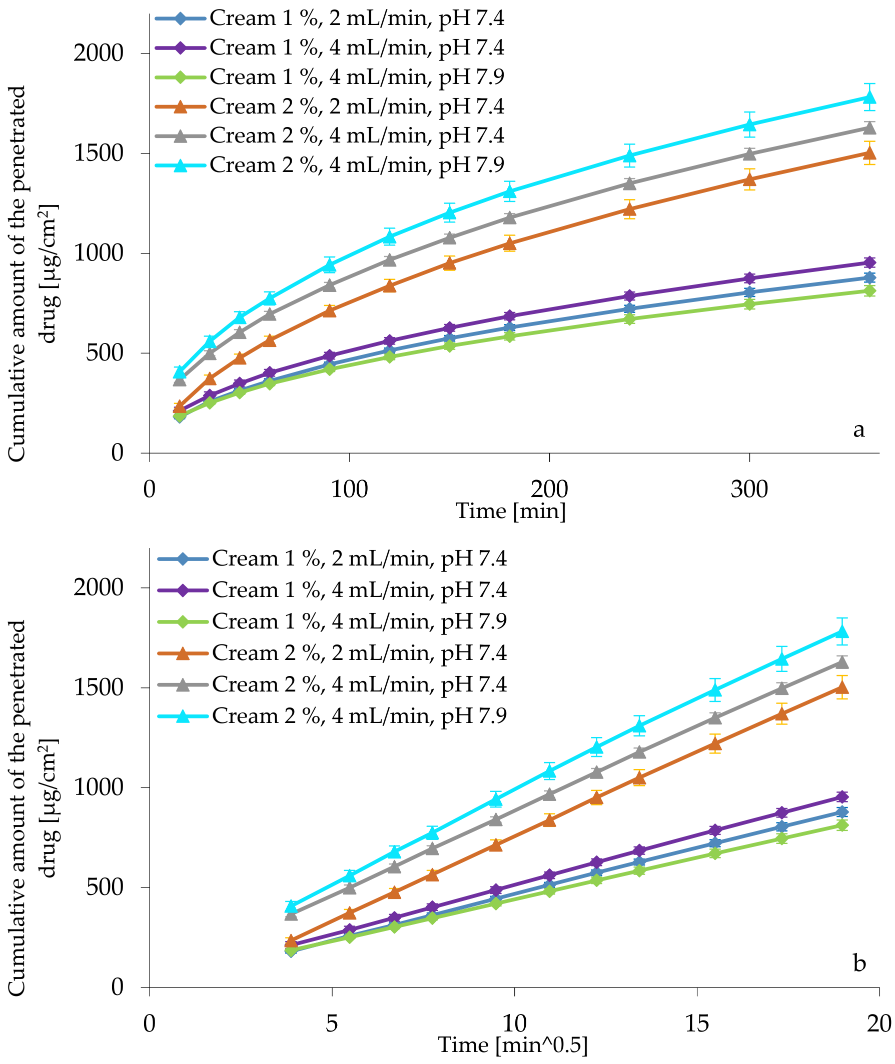

Figure S3. (**a**) Cumulative amount of diclofenac sodium penetrated through the MCE membrane plotted against time [min], (**b**) cumulative amount of diclofenac sodium penetrated through the MCE membrane plotted against square root of time [min^0.5]. Instrument: USP Apparatus IV with semisolid adapter. Receptor media: pH 7.4 and 7.9. Flow rate 2 and 4 mL/min. Product: Cream 1 % and 2 %. The data represent the mean ± standard error of the mean for six replicates.

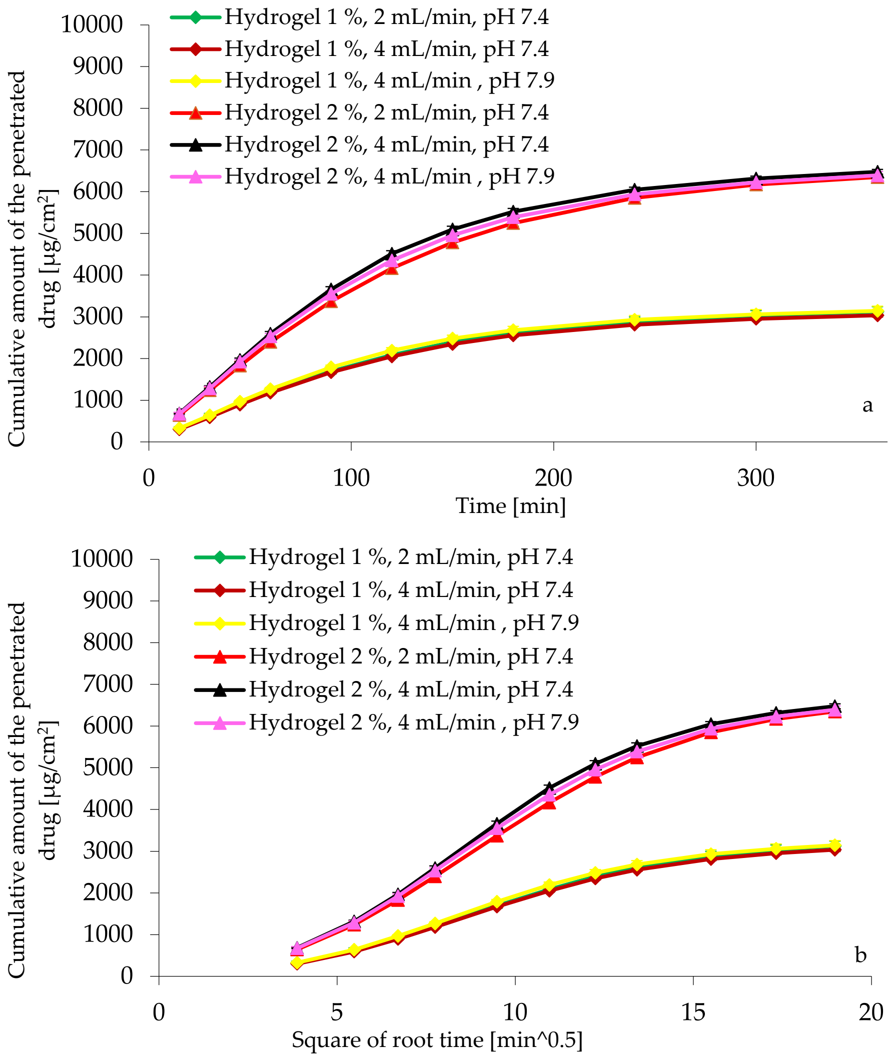

Figure S4. (**a**) Cumulative amount of diclofenac sodium penetrated through the MCE membrane plotted against time [min], (**b**) cumulative amount of diclofenac sodium penetrated through the MCE membrane plotted against square root of time [min^0.5]. Instrument: USP Apparatus IV with semisolid adapter. Receptor media: pH 7.4 and 7.9. Flow rate 2 and 4 mL/min Product: Hydrogel 1 % and 2 %. The data represent the mean ± standard error of the mean for six replicates.

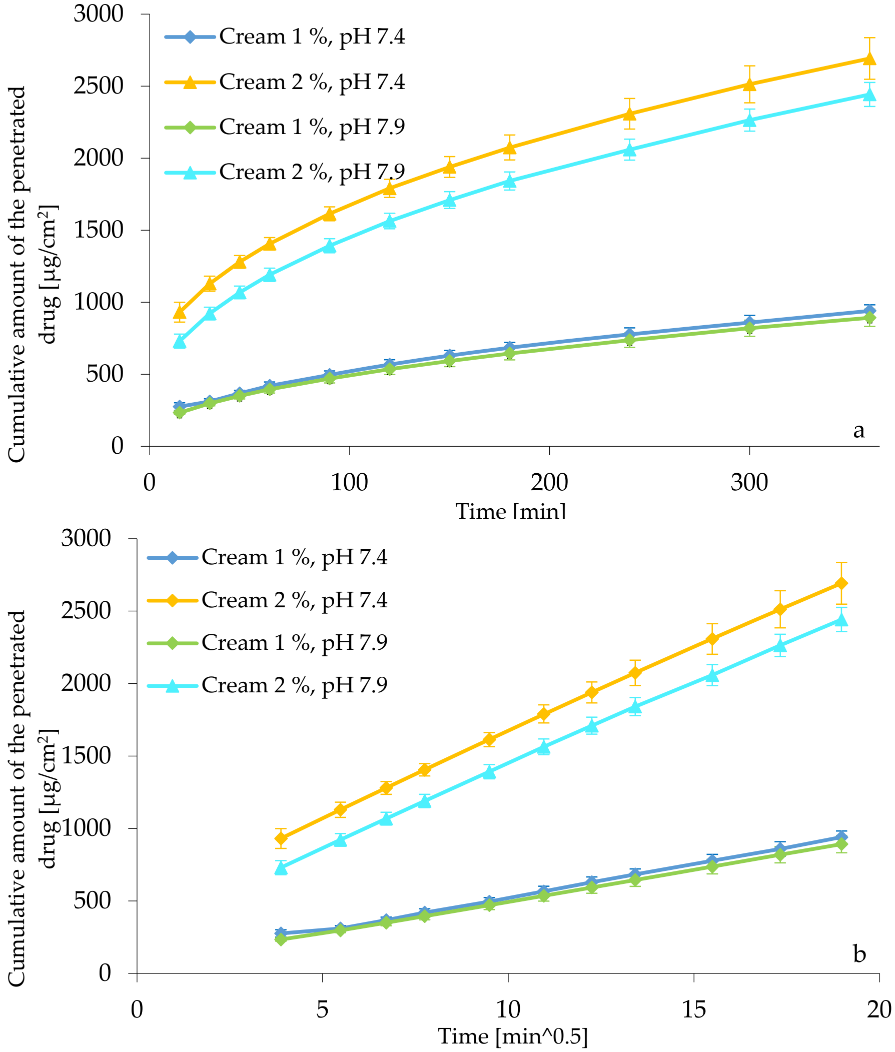

Figure S5. (**a**) Cumulative amount of diclofenac sodium penetrated through the MCE membrane plotted against time [min], (**b**) cumulative amount of diclofenac sodium penetrated through the MCE membrane plotted against square root of time [min^0.5]. Instrument: USP Apparatus II with immersion cell. Receptor media: pH 7.4 and 7.9. Product: Cream 1 % and 2 %. The data represent the mean ± standard error of the mean for six replicates.

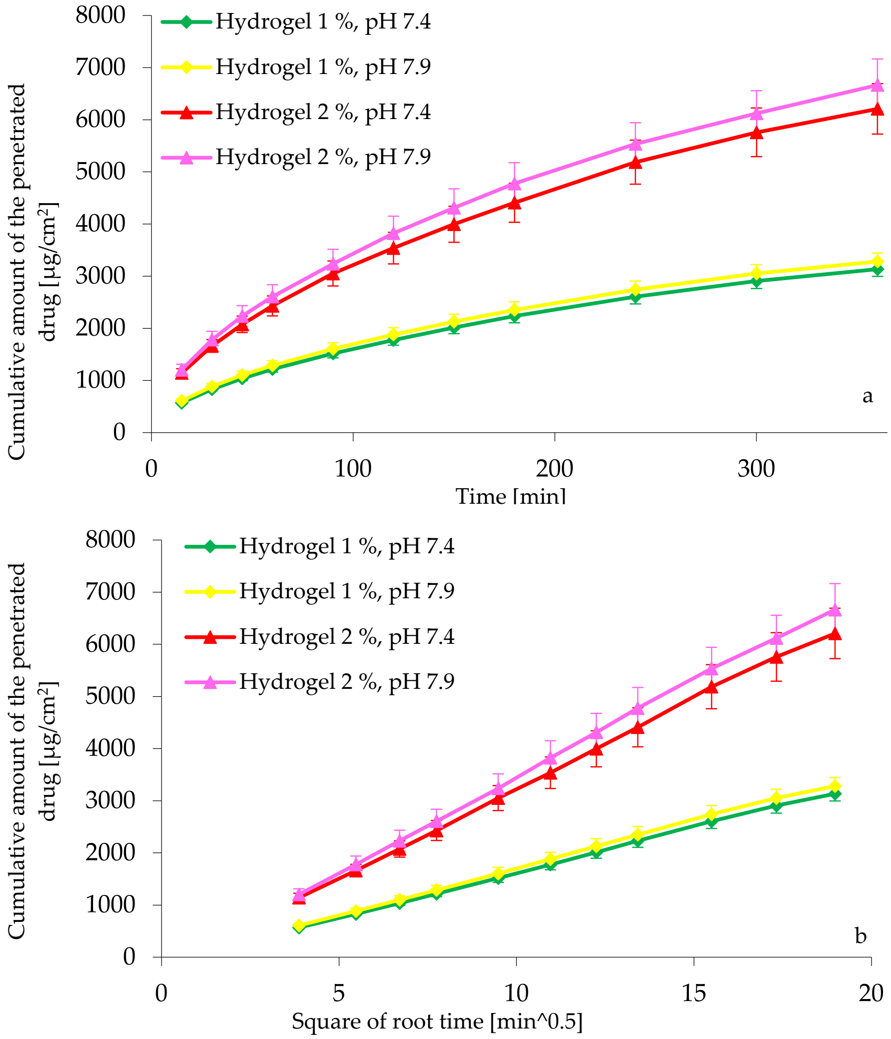

Figure S6. (**a**) Cumulative amount of diclofenac sodium penetrated through the MCE membrane plotted against time [min], (**b**) cumulative amount of diclofenac sodium penetrated through the MCE membrane plotted against square root of time [min^0.5]. Instrument: USP Apparatus II with immersion cell. Receptor media: pH 7.4 and pH 7.9. Product: Cream 1 % and 2 %. The data represent the mean ± standard error of the mean for six replicates.

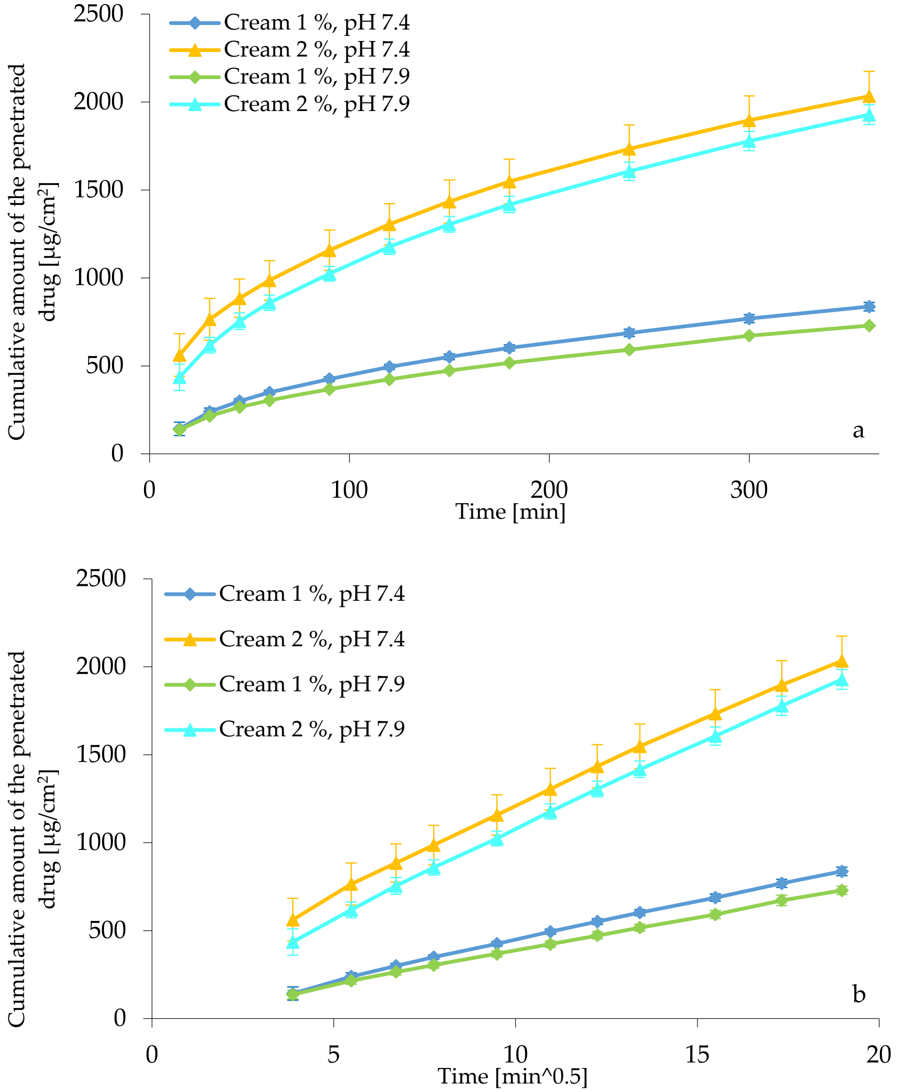

Figure S7. (**a**) Cumulative amount of diclofenac sodium penetrated through the MCE membrane plotted against time [min], (**b**) cumulative amount of diclofenac sodium penetrated through the MCE membrane plotted against square root of time [min^0.5]. Instrument: Static Vertical diffusion cell (Franz cell). Receptor media: pH 7.4 and 7.9. Product: Cream 1 % and 2 %. The data represent the mean ± standard error of the mean for six replicates.

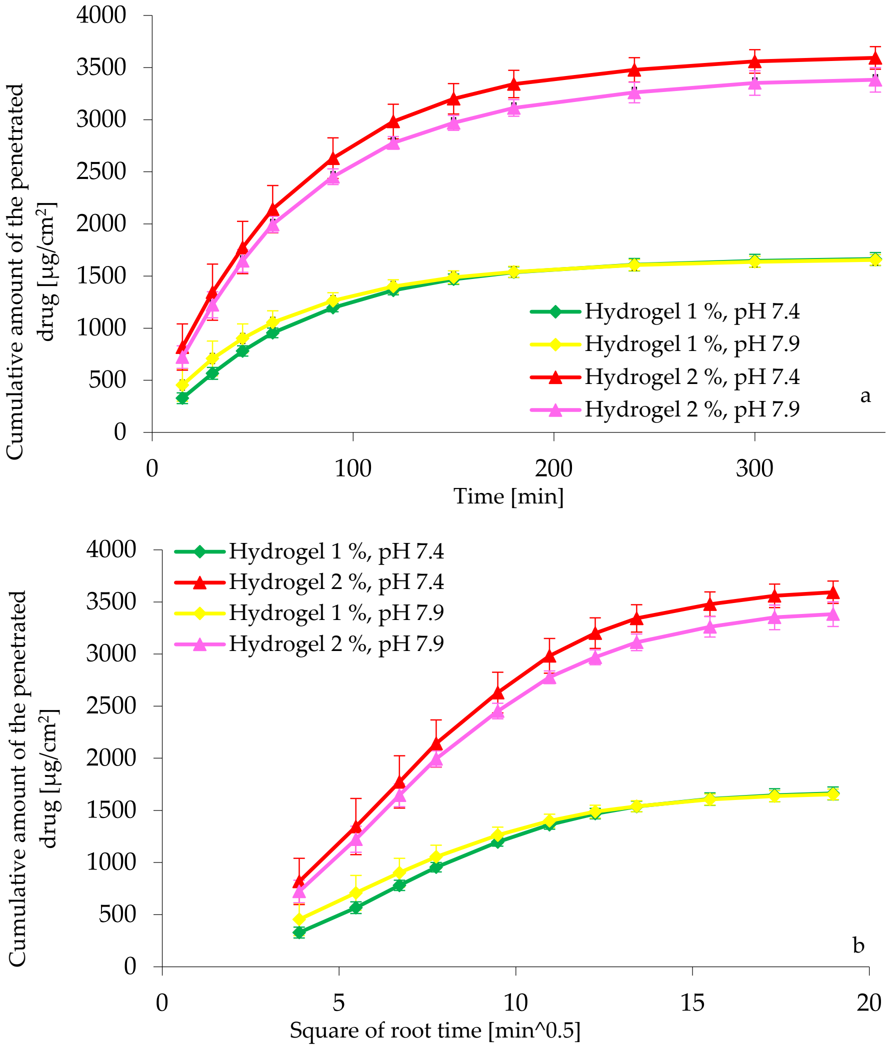

Figure S8. (**a**) Cumulative amount of diclofenac sodium penetrated through the MCE membrane plotted against time [min], (**b**) cumulative amount of diclofenac sodium penetrated through the MCE membrane plotted against square root of time [min^0.5]. Instrument: Static Vertical diffusion cell (Franz cell). Receptor media: pH 7.4 and 7.9. Product: Hydrogel 1 % and 2 %. The data represent the mean ± standard error of the mean for six replicates.

Table S1. *In vitro* release rates and fluxes of diclofenac sodium from cream 1% and 2 % at 6 hours measured with different methods with pH 7.4 medium.

Apparatus		Franz cell	Franz cell	USP IV with SSA	USP IV with SSA	USP IV with SSA	USP IV with SSA	USP II with immersion cell	USP II with immersion cell	FTDC	FTDC	FTDC	FTDC
API%		1%	2 %	1%	1%	2 %	2 %	1%	2%	1%	1%	2%	2%
pH		pH 7.4	pH 7.4	pH 7.4	pH 7.4	pH 7.4	pH 7.4	pH 7.4	pH 7.4	pH 7.4	pH 7.4	pH 7.4	pH 7.4
Flow rate (mL/min) or stirring speed (rpm)		400 rpm	400 rpm	2 mL/min	4 mL/min	2 mL/min	4 mL/min	250 rpm	250 rpm	2 mL/min	4 mL/min	2 mL/min	4 mL/min
IVRR at 6 h (%)	1	50.10	64.25	28.84	29.51	22.70	25.20	24.20	34.99	25.97	53.85	30.00	50.39
	2	50.65	62.89	28.58	31.42	23.74	26.47	26.67	36.08	36.52	69.86	28.06	37.25
	3	49.34	-	28.38	29.35	25.31	25.66	28.16	39.98	33.82	57.92	34.47	39.66
	4	50.27	58.35	28.18	30.09	23.28	25.52	25.63	35.25	27.06	55.74	45.42	40.14
	5	48.44	61.87	27.31	29.59	23.28	25.08	25.50	40.95	29.01	45.85	47.51	47.37
	6	46.93	59.18	27.26	30.19	22.68	25.77	26.39	40.75	**-**	**-**	**-**	**-**
	**Mean**	**49.29**	**61.31**	**28.09**	**30.02**	**23.50**	**25.62**	**26.09**	**38.00**	**30.47**	**56.64**	**37.09**	**42.96**
	**SD**	**1.40**	**2.49**	**0.66**	**0.76**	**0.97**	**0.49**	**1.33**	**2.85**	**4.52**	**8.68**	**8.90**	**5.62**
	**RSD%**	**2.83**	**4.06**	**2.36**	**2.53**	**4.13**	**1.93**	**5.09**	**7.49**	**14.85**	**15.33**	**23.99**	**13.07**
IVRR at 6 h (μg/cm²)	1	850.61	2181.78	905.54	944.80	1457.71	1631.09	916.09	2648.80	455.91	920.97	1046.68	1757.76
	2	859.89	2135.51	887.18	997.36	1523.73	1687.24	979.28	2649.58	643.21	1214.66	956.54	1269.78
	3	837.65	-	886.66	935.86	1611.07	1630.39	1002.24	2710.22	578.47	1016.90	1186.74	1365.42
	4	853.43	1981.30	889.19	959.57	1478.30	1610.62	912.16	2469.22	475.04	965.86	1548.41	1368.24
	5	822.38	2100.88	854.65	934.44	1487.63	1601.17	893.39	2776.14	501.02	781.50	1635.78	1631.15
	6	796.77	2009.37	847.09	952.19	1459.30	1610.59	939.28	2900.57	-	-	-	-
	**Mean**	**836.79**	**2081.77**	**878.38**	**954.04**	**1502.96**	**1628.51**	**940.41**	**2692.42**	**530.73**	**979.98**	**1274.83**	**1478.47**
	**SD**	**23.71**	**84.54**	**22.55**	**23.28**	**58.17**	**31.14**	**42.27**	**144.37**	**78.28**	**157.73**	**302.60**	**206.03**

Apparatus		Franz cell	Franz cell	USP IV with SSA	USP IV with SSA	USP IV with SSA	USP IV with SSA	USP II with immersion cell	USP II with immersion cell	FTDC	FTDC	FTDC	FTDC
API%		1%	2 %	1%	1%	2 %	2 %	1%	2%	1%	1%	2%	2%
pH		pH 7.4	pH 7.4	pH 7.4	pH 7.4	pH 7.4	pH 7.4	pH 7.4	pH 7.4	pH 7.4	pH 7.4	pH 7.4	pH 7.4
Flow rate (mL/min) or stirring speed (rpm)		400 rpm	400 rpm	2 mL/min	4 mL/min	2 mL/min	4 mL/min	250 rpm	250 rpm	2 mL/min	4 mL/min	2 mL/min	4 mL/min
	**RSD%**	**2.83**	**4.06**	**2.57**	**2.44**	**3.87**	**1.91**	**4.50**	**5.36**	**14.75**	**16.10**	**23.74**	**13.94**
Flux ($\mu g*cm^{-2}*min^{-0,5}$)	1	43.33	92.28	48.02	46.79	83.14	84.37	45.04	105.14	24.92	47.85	53.91	105.40
	2	45.41	97.10	48.08	52.94	86.01	88.01	48.05	112.24	26.48	61.32	54.72	67.13
	3	45.05	-	46.74	49.69	91.84	85.10	49.19	117.60	27.28	49.69	63.56	80.59
	4	45.76	101.63	46.28	50.25	82.06	84.39	44.03	98.59	24.74	54.27	85.05	93.87
	5	45.06	102.00	45.44	49.28	83.35	83.47	44.71	124.05	24.90	41.79	94.59	96.00
	6	40.20	94.22	45.19	50.41	83.35	85.19	47.14	129.50	-	-	-	-
	**Mean**	**44.13**	**97.45**	**46.63**	**49.89**	**84.96**	**85.09**	**46.36**	**114.52**	**25.67**	**50.98**	**70.37**	**88.60**
	**SD**	**2.10**	**4.34**	**1.24**	**1.99**	**3.61**	**1.56**	**2.07**	**11.59**	**1.15**	**7.31**	**18.47**	**14.92**
	**RSD%**	**4.76**	**4.46**	**2.66**	**3.98**	**4.25**	**1.83**	**4.46**	**10.12**	**4.47**	**14.33**	**26.25**	**16.84**

Table S2. *In vitro* release rates and fluxes of diclofenac sodium from hydrogel 1% and 2 % at 6 hours measured with different methods with pH 7.4 medium.

Apparatus		Franz cell	Franz cell	USP IV with SSA	USP IV with SSA	USP IV with SSA	USP IV with SSA	USP II with immersion cell	USP II with immersion cell	FTDC	FTDC	FTDC	FTDC
API%		1%	2 %	1%	1%	2 %	2 %	1%	2%	1%	1%	2%	2%
pH		pH 7.4	pH 7.4	pH 7.4	pH 7.4	pH 7.4	pH 7.4	pH 7.4	pH 7.4	pH 7.4	pH 7.4	pH 7.4	pH 7.4
Flow rate (mL/min) or stirring speed (rpm)		400 rpm	400 rpm	2 mL/min	4 mL/min	2 mL/min	4 mL/min	250 rpm	250 rpm	2 mL/min	4 mL/min	2 mL/min	4 mL/min
IVRR at 6 h (%)	1	92.75	89.11	98.70	93.17	98.30	99.84	80.70	97.85	96.02	101.93	82.13	97.89
	2	95.52	86.99	98.58	96.44	99.78	100.70	87.62	82.15	91.52	98.00	81.49	98.04
	3	94.49	92.76	100.65	94.35	98.56	101.15	87.85	81.58	92.82	113.34	84.26	99.38
	4	95.99	93.42	103.50	93.92	99.09	100.90	88.31	94.34	91.19	101.21	81.55	97.41
	5	91.02	90.20	100.83	94.30	99.10	101.45	87.86	78.49	92.79	108.59	99.05	100.95
	6	84.59	97.94	91.47	93.17	97.08	99.38	86.49	86.76	-	-	-	-
	**Mean**	**92.39**	**91.74**	**98.95**	**94.23**	**98.65**	**100.57**	**86.47**	**86.86**	**92.87**	**104.61**	**85.70**	**98.74**
	**SD**	**4.24**	**3.85**	**4.08**	**1.20**	**0.93**	**0.80**	**2.89**	**7.71**	**1.91**	**6.21**	**7.55**	**1.44**
	**RSD%**	**4.59**	**4.20**	**4.12**	**1.28**	**0.94**	**0.80**	**3.35**	**8.87**	**2.06**	**5.94**	**8.81**	**1.45**
IVRR at 6 h (μg/cm²)	1	1627.20	3631.14	3086.13	2992.63	6347.96	6429.02	2872.26	6634.17	1658.57	1783.7	2800.04	3303.95
	2	1675.75	3741.59	3087.21	3114.36	6461.93	6449.25	3267.01	5940.61	1612.07	1703.8	2778.15	3475.94
	3	1764.70	3674.72	3209.02	3020.51	6334.09	6555.98	3226.02	5715.14	1603.29	1983.4	2872.41	3455.76
	4	1684.05	3489.22	3212.61	3046.20	6332.82	6438.57	3143.37	6715.88	1580.26	1759.6	2872.64	3387.31
	5	1648.33	3471.07	3227.52	3022.51	6362.45	6544.63	3176.94	5676.07	1576.45	1912.6	3511.86	3464.32
	6	1579.71	3547.28	2924.78	3013.63	6257.43	6445.74	3127.50	6568.11	-	-	-	-
	**Mean**	**1663.29**	**3592.50**	**3124.55**	**3034.97**	**6349.44**	**6477.20**	**3135.51**	**6208.33**	**1606.13**	**1828.62**	**2967.02**	**3417.46**
	**SD**	**62.26**	**107.65**	**116.85**	**42.52**	**66.03**	**57.16**	**139.02**	**483.03**	**32.95**	**115.51**	**307.51**	**72.24**
	**RSD%**	**3.74**	**3.00**	**3.74**	**1.40**	**1.04**	**0.88**	**4.43**	**7.78**	**2.05**	**6.32**	**10.36**	**2.11**

Apparatus		Franz cell	Franz cell	USP IV with SSA	USP IV with SSA	USP IV with SSA	USP IV with SSA	USP II with immersion cell	USP II with immersion cell	FTDC	FTDC	FTDC	FTDC
API%		1%	2 %	1%	1%	2 %	2 %	1%	2%	1%	1%	2%	2%
pH		pH 7.4	pH 7.4	pH 7.4	pH 7.4	pH 7.4	pH 7.4	pH 7.4	pH 7.4	pH 7.4	pH 7.4	pH 7.4	pH 7.4
Flow rate (mL/min) or stirring speed (rpm)		400 rpm	400 rpm	2 mL/min	4 mL/min	2 mL/min	4 mL/min	250 rpm	250 rpm	2 mL/min	4 mL/min	2 mL/min	4 mL/min
Flux (µg*cm-2*min-0,5)	1	156.14	326.88	264.12	286.09	540.86	618.28	153.61	375.11	174.02	164.71	317.42	405.64
	2	164.59	348.80	267.91	280.99	563.08	659.90	185.65	337.47	173.66	178.81	301.12	538.02
	3	170.02	344.78	273.39	291.83	573.73	624.32	186.11	309.90	176.40	195.66	311.87	410.90
	4	167.71	337.83	272.33	342.25	627.29	613.69	180.09	372.07	169.56	199.47	313.78	407.95
	5	169.33	341.06	265.71	292.97	574.12	627.30	182.44	301.71	171.10	184.32	424.62	431.16
	6	146.99	350.09	265.23	288.41	543.94	614.00	178.41	381.09	-	-	-	-
	**Mean**	**162.46**	**341.57**	**268.11**	**297.09**	**570.50**	**626.25**	**177.72**	**346.22**	**172.95**	**184.59**	**333.76**	**438.73**
	**SD**	**9.12**	**8.55**	**3.89**	**22.54**	**31.26**	**17.37**	**12.19**	**34.92**	**2.67**	**13.90**	**51.15**	**56.42**
	**RSD%**	**5.61**	**2.50**	**1.45**	**7.59**	**5.48**	**2.77**	**6.86**	**10.08**	**1.54**	**7.52**	**15.33**	**12.60**

Table S3. Results of accuracy measurement at a nominal concentration of 100% using cream matrix.

Name of the apparatus	Accuracy<br>%	Mean of accuracy<br>%	SD of accuracy<br>%	RSD of accuracy<br>%
Franz cell	96.64<br>96.88<br>96.48<br>96.45<br>96.77<br>96.26	96.58	0.23	0.23
USP II, immersion cell	101.60<br>101.13<br>100.94<br>101.62<br>101.74<br>98.62	100.94	1.18	1.17
USP IV, SSA	99.11<br>99.18<br>98.96<br>99.17<br>99.09<br>98.74	99.04	0.17	0.17

Table S4. Results of accuracy measurement at a nominal concentration of 100% using hydrogel matrix.

Name of the apparatus	Accuracy<br>%	Mean of accuracy<br>%	SD of accuracy<br>%	RSD of accuracy<br>%
Franz cell	94.51<br>93.78<br>95.98<br>94.72<br>94.15<br>94.70	94.64	0.75	0.79
USP II, immersion cell	103.53<br>101.49<br>100.98	102.00	1.35	1.32
USP IV, SSA	96.91<br>96.83<br>97.26<br>97.21<br>97.15<br>97.14	97.08	0.17	0.18

Table 5. IVRR and fluxes of diclofenac sodium from 1% and 2 % cream measured for 6 hours using different methods with pH 7.9 medium.

Apparatus		Franz cell	Franz cell	USP IV with SSA	USP IV with SSA	USP II with immersion cell	USP II with immersion cell
API (%)		1%	2 %	1%	2 %	1%	2%
pH		pH 7.9	pH 7.9	pH 7.9	pH 7.9	pH 7.9	pH 7.9
Flow rate (mL/min) or stirring speed (rpm)		400 rpm	400 rpm	4 ml/min	4 ml/min	250 rpm	250 rpm
IVRR at 6 h (%)	1	45.09	57.91	24.43	26.14	24.45	34.37
	2	48.07	55.87	27.40	28.76	24.71	34.85
	3	44.43	55.94	27.03	28.82	25.57	37.48
	4	45.86	54.45	26.38	27.85	23.33	33.90
	5	39.56	59.04	26.69	28.17	26.58	36.27
	6	37.92	57.51	25.98	28.30	25.72	36.44
	**Mean**	**43.49**	**56.79**	**26.32**	**28.01**	**25.06**	**35.55**
	**SD**	**3.91**	**1.66**	**1.05**	**0.98**	**1.14**	**1.39**
	**RSD%**	**9.00**	**2.93**	**3.99**	**3.52**	**4.54**	**3.91**
IVRR at 6 h (μg/cm^2)	1	765.47	1966.24	767.19	1676.51	856.58	2407.59
	2	734.49	1897.21	841.68	1847.06	865.69	2520.54
	3	704.09	1899.64	828.30	1862.71	866.70	2498.86
	4	700.79	1848.91	815.16	1783.82	830.47	2298.52
	5	738.79	2004.79	822.01	1755.37	976.26	2500.31
	6	729.61	1952.70	801.38	1767.55	958.92	2429.40
	**Mean**	**728.88**	**1928.25**	**812.62**	**1782.17**	**892.44**	**2442.54**
	**SD**	**23.97**	**56.53**	**25.98**	**67.52**	**59.92**	**83.34**
	**RSD%**	**3.29**	**2.93**	**3.20**	**3.79**	**6.71**	**3.41**
Flux (μg*cm^{-2}*min$^{-0.5}$)	1	38.69	97.22	38.77	87.85	41.96	104.79
	2	37.27	89.35	43.28	95.47	43.60	116.65
	3	35.75	95.90	43.17	98.60	42.84	114.82
	4	37.70	95.20	42.26	92.89	41.13	104.54
	5	39.67	101.98	43.13	92.81	48.89	111.86
	6	39.05	101.02	41.04	93.00	47.87	114.26
	**Mean**	**38.02**	**96.78**	**41.94**	**93.44**	**44.38**	**111.15**
	**SD**	**1.42**	**4.55**	**1.77**	**3.55**	**3.22**	**5.25**
	**RSD%**	**3.73**	**4.70**	**4.23**	**3.79**	**7.26**	**4.73**

Table S6. IVRR and fluxes of diclofenac sodium from 1% and 2 % hydrogel measured for 6 hours using different methods with pH 7.9 medium.

Apparatus		Franz cell	Franz cell	USP IV with SSA	USP IV with SSA	USP II with immersion cell	USP II with immersion cell
API (%)		1%	2 %	1%	2 %	1%	2%
pH		pH 7.9	pH 7.9	pH 7.9	pH 7.9	pH 7.9	pH 7.9
Flow rate (mL/min) or stirring speed (rpm)		400 rpm	400 rpm	4 mL/min	4 mL/min	250 rpm	250 rpm
IVRR at 6 h (%)	1	95.06	98.12	99.33	99.09	89.10	90.91
	2	94.98	96.55	98.64	101.27	90.39	90.93
	3	102.75	98.42	99.98	100.09	88.26	86.81
	4	94.34	99.59	91.67	98.62	87.54	86.74
	5	94.75	101.82	100.87	99.56	86.44	87.37
	6	99.78	99.85	98.33	98.14	86.09	89.14
	**Mean**	**96.94**	**99.06**	**98.14**	**99.46**	**87.97**	**88.65**
	**SD**	**3.48**	**1.80**	**3.30**	**1.12**	**1.63**	**1.96**
	**RSD%**	**3.59**	**1.81**	**3.36**	**1.13**	**1.85**	**2.21**
IVRR at 6 h (μg/cm^2)	1	1613.93	3331.71	3067.01	6429.19	3523.89	6676.81
	2	1666.24	3278.47	3026.14	6524.75	3421.65	7294.96
	3	1744.46	3230.43	3120.83	6447.63	3191.40	6179.97
	4	1601.69	3494.31	3086.53	6320.37	3264.14	6272.47
	5	1662.34	3457.41	3064.98	6363.96	3076.84	6318.42
	6	1637.52	3503.58	3001.20	6266.47	3209.96	7252.47
	**Mean**	**1654.36**	**3382.65**	**3061.12**	**6392.06**	**3281.31**	**6665.85**
	**SD**	**51.02**	**117.73**	**42.60**	**93.57**	**163.59**	**500.31**
	**RSD%**	**3.08**	**3.48**	**1.39**	**1.46**	**4.99**	**7.51**
Flux (μg*cm^{-2}*min$^{-0.5}$)	1	134.41	320.16	284.95	583.77	201.41	382.52
	2	138.16	323.03	280.74	529.59	195.21	413.38
	3	161.57	310.08	294.69	563.12	182.16	348.09
	4	162.30	340.68	268.55	594.92	187.68	358.06
	5	170.65	336.01	296.63	514.03	171.08	355.39
	6	165.64	346.28	291.72	615.60	178.02	403.65
	**Mean**	**155.46**	**329.37**	**286.21**	**566.84**	**185.93**	**376.85**
	**SD**	**15.24**	**13.82**	**10.53**	**39.10**	**11.19**	**27.30**
	**RSD%**	**9.80**	**4.20**	**3.68**	**6.90**	**6.02**	**7.24**
